# A post-hoc analysis of the comparative efficacy of canagliflozin and glimepiride in the attainment of type 2 diabetes-related quality measures

**DOI:** 10.1186/s12913-016-1607-z

**Published:** 2016-08-05

**Authors:** Charmi A. Patel, Robert A. Bailey, Ujjwala Vijapurkar, Gary Meininger, Lawrence Blonde

**Affiliations:** 1 Janssen Scientific Affairs, LLC, 1000 Route 202 South, Raritan, NJ 08869 USA; 2 Janssen Research and Development, LLC, 920 US Route 202 South, Raritan, NJ 08869 USA; 3 Ochsner Diabetes Clinical Research Unit, Frank Riddick Diabetes Institute, Department of Endocrinology, Ochsner Medical Center, 1515 Jefferson Highway, New Orleans, LA 70121 USA

**Keywords:** Canagliflozin, Glimepiride, Quality measures, Post-hoc analysis, Phase 3, Glycemic control, Blood pressure, Body weight, A1C

## Abstract

**Background:**

The randomized, double-blind CANTATA-SU (CANagliflozin Treatment And Trial Analysis Sulfonyl Urea) clinical trial compared the use of canagliflozin (100 mg or 300 mg) and maximally tolerated glimepiride (6–8 mg) over 104 weeks as add-on therapy for patients with type 2 diabetes mellitus (T2DM) inadequately controlled with metformin. Compared with glimepiride, canagliflozin use was associated with durable reductions in glycated hemoglobin (A1C), blood pressure (BP), and body weight. The aim of this post-hoc analysis of the CANTATA-SU trial was to assess the comparative efficacy of canagliflozin and glimepiride in the attainment of recently updated diabetes-related quality measures (QMs) for up to 104 weeks of treatment.

**Methods:**

This post-hoc analysis evaluated the proportions of patients achieving individual diabetes-related QMs using data from the randomized, double-blind, Phase 3 CANTATA-SU trial. Change in A1C from baseline, and proportions of the study population achieving QMs: A1C <7.0 %, <8.0 %, and >9.0 % were assessed. Secondary endpoints included change in BP from baseline, and the proportions of the study population achieving QMs related to BP and body weight.

**Results:**

The proportions of patients in the canagliflozin 100 mg, canagliflozin 300 mg, and glimepiride groups meeting criteria for all QMs were similar at baseline. At 52 and 104 weeks of treatment, canagliflozin 100 mg and canagliflozin 300 mg provided better or similar reductions in A1C from baseline and achievement of glycemic control QMs compared with glimepiride. At 52 and 104 weeks of treatment, the attainment of QMs related to reductions in body weight and BP all favored canagliflozin compared with glimepiride. Canagliflozin was associated with lower incidence of documented hypoglycemia and severe hypoglycemia compared with glimepiride.

**Conclusions:**

Using the recently adjusted and currently accepted diabetes-related QMs, this analysis observed superior glycemic control with canagliflozin compared with maximally tolerated glimepiride in patients with T2DM who were previously poorly controlled on metformin monotherapy. Compared with maximally tolerated glimepiride, canagliflozin resulted in better achievement of diabetes-related QMs related to weight loss and BP, and was associated with lower incidences of hypoglycemic events.

**Trial registration:**

Clinical trial registry name: CANagliflozin Treatment And Trial Analysis-Sulfonylurea (CANTATA-SU) SGLT2 Add-on to Metformin Versus Glimepiride.

Clinical trial registration number: NCT00968812, registered August 28, 2009.

## Background

The attainment of good glycemic control is a major goal in the treatment of type 2 diabetes mellitus (T2DM). Guidelines issued jointly by the American Diabetes Association (ADA) and European Association for the Study of Diabetes (EASD), and those from the American Association of Clinical Endocrinologists and the American College of Endocrinology (AACE/ACE), all emphasize the importance of achieving glycemic goals, as measured by glycated hemoglobin (A1C) but stress the need for the individualization of treatment to meet the needs of each specific patient [1–7]. In addition to the use of clinical guideline-based glycemic goals, there has been an increased focus in recent years on the use of quality measures (QMs) as benchmarks to evaluate patient outcomes, and as reporting tools aimed at improving the health of the overall population and reducing healthcare costs [8]. With estimated diabetes-related healthcare costs amounting to $322 billion in the United States (US) in 2012 [9], there is a clear need for the measurement of outcomes associated with improved health while at the same time reducing the financial burden. The National Committee for Quality Assurance has established the HEDIS (Healthcare Effectiveness Data and Information Set) Comprehensive Care measures, which include measures related to T2DM [10]. HEDIS measures allow for evidence-based comparisons of quality performance across different health plans. Other organizations have established their own QMs for diabetes management, including the Health Resources and Services Administration’s Health Disparities Collaborative, Better Health’s Clinical Advisory Committee, and the Centers for Medicare and Medicaid [11–13].

In April 2015, a number of changes were made to the HEDIS diabetes-related QMs to bring them into line with the recent clinical guidelines [14]. These changes included keeping the A1C to <7.0 % goal for a selected population without comorbid conditions and looking more closely at patients with A1C <8.0 % (considered to be controlled) as well as patients with A1C >9.0 % (considered to be poorly controlled); redefining blood pressure (BP) control as <140/90 mm Hg, rather than <140/80 mm Hg; and removing low density lipoprotein-cholesterol (LDL-C) screening and the LDL-C target of <100 mg/dL. Modifying the BP control measure aligns the QMs with the most recent hypertension guidelines issued by the Eighth Joint National Committee [15]. The changes regarding LDL-C align the QMs with the latest American College of Cardiology/American Heart Association (ACC/AHA) Task Force on Practice Guidelines [15].

In combination with lifestyle changes, the current ADA/EASD guidelines for the treatment of T2DM advocate a patient-centered approach and endorse the value of different classes of antihyperglycemic agents (AHAs), in addition to metformin, as a component of dual- and triple-drug regimens [1]. Sulfonylureas have been widely used for many years in the management of T2DM because of their low cost and long-term clinical experience. However, they are associated with an increased risk of hypoglycemia and weight gain, and have a relatively low durability compared with some of the other AHAs [2]. The AACE/ACE algorithm in particular emphasizes the use of agents with a low risk of hypoglycemia and/or weight gain [6, 7].

Sodium-glucose co-transporter 2 (SGLT2) inhibitors are a new class of AHAs which reduce blood glucose by targeting the kidney to increase urinary glucose excretion. These agents were recently included in the ADA/EASD Algorithm for antihyperglycemic therapy in T2DM [2, 3] and the 2015 AACE/ACE Comprehensive Diabetes Management Algorithm [6, 7]. In addition to improving glycemic control, SGLT2 inhibitors have been shown to reduce body weight and systolic blood pressure (SBP) [16]. Due to their insulin-independent mechanism of action, SGLT2 inhibitors are also associated with a low risk of hypoglycemic episodes when they are used as monotherapy or in combination with other AHAs not associated with a high risk of hypoglycemia (sulfonylureas and insulins can induce hypoglycemia) [17, 18]. Canagliflozin was the first SGLT2 inhibitor approved for use in the US for the improvement of glycemic control in adult patients with T2DM [19].

To date, very few studies have compared the attainment of QMs between different classes of AHAs, and particularly between the older and newer classes of agents. Results of a previous post-hoc analysis suggested that canagliflozin was associated with comparable or superior attainment of QMs when compared with sitagliptin (100 mg) [20]. Additional studies that assess the achievement of QMs amongst the various AHAs are needed to evaluate the quality of care received by patients with T2DM. The randomized, double-blind CANTATA-SU (CANagliflozin Treatment And Trial Analysis Sulfonyl Urea) clinical trial compared the efficacy, safety and tolerability of canagliflozin (100 mg and 300 mg) and maximally tolerated glimepiride administered over 104 weeks as add-on therapy for patients with T2DM inadequately controlled with metformin. Compared with glimepiride, both doses of canagliflozin were associated with durable reductions in A1C, body weight and SBP [21, 22]. At 52 weeks, canagliflozin 100 mg was shown to be non-inferior to glimepiride, and canagliflozin 300 mg was found to be superior to glimepiride in lowering A1C [21]. These effects were found to be durable and were maintained at 104 weeks [22]. Additionally, over 104 weeks, canagliflozin 100 mg and canagliflozin 300 mg were associated with lower incidences of documented hypoglycemia than glimepiride [22]. The aim of the present analysis was to assess the comparative efficacy of canagliflozin 100 mg, canagliflozin 300 mg, and maximally tolerated glimepiride (6–8 mg) in the attainment of the recently updated diabetes-related QMs over 104 weeks of treatment, using data obtained from the CANTATA-SU trial.

## Methods

### Patient population

This analysis used data from the CANTATA-SU trial, details of which (study, inclusion and exclusion criteria, outcomes) have been previously reported [21, 22].

Patients (*N* = 1,450) received canagliflozin 100 mg, canagliflozin 300 mg, or glimepiride during 104 weeks of treatment. Glimepiride could be titrated multiple times and at any point up to 6 or 8 mg/day based on the maximum approved dose in each country. The total treatment phase consisted of a 52-week core double-blind period followed by a 52-week extension double-blind period. The study compared the efficacy of 2 doses of canagliflozin (100 mg and 300 mg) and glimepiride, with respect to the change from baseline in A1C, SBP, diastolic blood pressure (DBP), body weight, fasting plasma glucose, and fasting lipids, among other parameters [21, 22].

### Study measures and statistical analyses

#### Efficacy analyses

Efficacy data from the CANTATA-SU trial were used to evaluate the proportions of patients achieving individual diabetes-related QMs of glycemic control, BP control, and body mass index (BMI)/body weight at baseline, 52 weeks, and 104 weeks.

The initial endpoints analyzed were change in A1C from baseline, and patients achieving A1C <7.0 % or <8.0 %, or who were poorly controlled (>9.0 %) at Week 52. Secondary endpoints in this analysis included: change in BP (SBP and DBP) from baseline, patients achieving BP <140/90 mm Hg, change in BMI from baseline, patients achieving BMI ≤30 kg/m^2^, and patients with BMI ≥25 kg/m^2^ at baseline who lost ≥10 lb (4.5 kg).

An analysis of covariance (ANCOVA) model with treatment, stratification factors and country as fixed effects, and baseline values as covariates, was used to evaluate changes or percent changes from baseline at Week 52 and Week 104 in the continuous efficacy variables. Odds ratio (OR) and 95 % confidence intervals (CIs) were calculated for the proportion of patients achieving treatment goals based on a logistic model with treatment, stratification factors, and country as fixed effects, and corresponding baseline values as covariates. The analyses were based on the modified intent-to-treat (mITT) analysis set, which included all randomized patients who took at least one dose of double-blind study medication. The post-baseline last observation carried forward (LOCF) imputation method was applied when values for Week 52 or Week 104 were missing. After Day 1 and during the 104-week double-blind treatment phase, glycemic rescue medication (pioglitazone) was administered to patients on maximum background therapy dose level (maximum allowed dose level of glimepiride) who met pre-specified, stringent glycemic rescue criteria [21]. For patients placed on rescue medication during the study, only the data prior to the initiation of rescue medication were used for the analyses.

#### Safety analyses

The incidence (i.e., number and percent of patients with 1 or more events in each category) of adverse events (AEs), serious AEs, AEs leading to discontinuation, cardiovascular AEs, and AEs related to study drug were summarized by treatment group and have been reported previously [21, 22].

The safety analysis included the incidence of hypoglycemia, genital mycotic infections (GMIs), urinary tract infections (UTIs), osmotic diuresis-related AEs, and volume-depletion AEs, as well as proportions of patients receiving antihypertensive agents. All data, regardless of the initiation of rescue medication, were used to perform the safety analyses for overall and specific AEs, while data prior to the initiation of rescue medication were used for hypoglycemia only. Results of the safety analysis are given for the core (baseline through 52 weeks) and for the entire treatment period (baseline through 104 weeks).

## Results

### Patients

Of the 1,452 patients randomized to study treatment, 1,450 were included in the modified intent-to-treat (mITT analysis set), with 483 patients in the canagliflozin 100 mg, 485 patients in the canagliflozin 300 mg, and 482 patients in the glimepiride groups. The mean (standard deviation, SD) maximum dose achieved with glimepiride was 5.6 (2.3) mg at Week 52, and 5.8 (2.2) mg at Week 104.

Overall, 67.6 % of patients completed the entire 104-week treatment period. The most common reasons for discontinuation across all treatment groups included AEs (7.5 % of patients), other (7.0 % of patients), and withdrawal of consent (4.5 % of patients). Efficacy and safety analyses were both performed using the mITT analysis set.

### Baseline demographics and disease characteristics

Baseline demographics for the overall population are shown in Table 1. Patients had similar characteristics across different treatment groups. The mean age of the patient population was 56 years, and 52 % of patients were male. Consistent with the regions of the world in which patients were recruited, 67 % of the patients were white, 20 % were Asian, 17 % of patients were Hispanic or Latino, and 4 % were Black /African American. Of the US residents who participated in the study, 16 % were Black/African American. At baseline, the mean body weight was 86.6 kg and mean baseline BMI was 31.0 kg/m^2^; approximately 54 % of patients were defined as obese (BMI ≥30 kg/m^2^) in accordance to the National Institutes of Health criteria [23]. Baseline diabetes disease characteristics of the overall population were also generally similar across the treatment groups (Table 2). All patients had mild-to-moderate hyperglycemia at baseline and a mean duration of diabetes of 6.6 years.

**Table 1:** Baseline demographic and anthropometric characteristics in the overall population

	CANA 100 mg (*N* = 483)	CANA 300 mg (*N* = 485)	GLIM (*N* = 482)
Sex, n (%)			
Male	252 (52.2)	241 (49.7)	263 (54.6)
Female	231 (47.8)	244 (50.3)	219 (45.4)
Age (years)			
Category, n (%)			
<65	397 (82.2)	411 (84.7)	399 (82.8)
≥65	86 (17.8)	74 (15.3)	83 (17.2)
Mean (SD)	56.4 (9.49)	55.8 (9.17)	56.3 (9.01)
Race, n (%)			
White	323 (66.9)	333 (68.7)	322 (66.8)
Black/African American	20 (4.1)	18 (3.7)	22 (4.6)
Asian	99 (20.5)	93 (19.2)	93 (19.3)
Other	41 (8.4)	41 (8.5)	45 (9.3)
Ethnicity, n (%)			
Hispanic/Latino	86 (17.8)	80 (16.5)	76 (15.8)
Not Hispanic/Latino	395 (81.8)	404 (83.3)	403 (83.6)
Not reported	1 (0.2)	1 (0.2)	1 (0.2)
Unknown	1 (0.2)	0 (0)	2 (0.4)
Body weight (kg)			
Mean (SD)	86.9 (20.06)	86.6 (19.48)	86.5 (19.82)
BMI (kg/m^2^), n (%)			
<30	215 (44.5)	224 (46.2)	234 (48.5)
≥30	268 (55.5)	261 (53.8)	248 (51.5)
Mean (SD)	31.0 (5.29)	31.2 (5.39)	30.9 (5.54)

*BMI* body mass index, *CANA* canagliflozin, *GLIM* glimepiride, *SD* standard deviation

**Table 2:** Baseline diabetes characteristics in the overall population

	CANA 100 mg (*N* = 483)	CANA 300 mg (*N* = 485)	GLIM (*N* = 482)
Baseline A1C, n (%)			
<7.0 %	59 (12.2)	71 (14.6)	65 (13.5)
<8.0 % (good control)	294 (60.9)	294 (60.6)	290 (60.2)
>9.0 % (poor control)	31 (6.4)	34 (7.0)	34 (7.1)
Mean (SD)	7.8 (0.78)	7.8 (0.78)	7.8 (0.80)
Baseline FPG (mmol/L)			
Mean (SD)	9.2 (2.07)	9.1 (2.01)	9.2 (2.11)
Duration of diabetes (years)			
Mean (SD)	6.5 (5.47)	6.7 (5.50)	6.6 (5.02)
Patients with microvascular complications, n (%)			
N	93	87	90
Neuropathy	75 (15.5)	62 (12.8)	67 (13.9)
Retinopathy	26 (5.4)	37 (7.6)	27 (5.6)
Nephropathy	18 (3.7)	15 (3.1)	16 (3.3)
Patients with number of microvascular complications, n (%)			
0	390 (80.7)	398 (82.1)	392 (81.3)
1	71 (14.7)	65 (13.4)	72 (14.9)
2	18 (3.7)	17 (3.5)	16 (3.3)
3	4 (0.8)	5 (1.0)	2 (0.4)
Baseline eGFR (mL/min/1.73 m^2^)			
N	483	485	481
Category, n (%)			
<60	15 (3.1)	13 (2.7)	10 (2.1)
60– < 90	232 (48.0)	232 (47.8)	251 (52.1)
≥90	236 (48.9)	240 (49.5)	220 (45.6)
Mean (SD)	89.7 (19.28)	91.4 (19.36)	89.5 (17.48)

*A1C* glycated hemoglobin, *AHA* antihyperglycemic agent, *CANA* canagliflozin, *eGFR* estimated glomerular filtration rate, *FPG* fasting plasma glucose, *GLIM* glimepiride, *SD* standard deviation

### Efficacy/quality measure attainment

#### Efficacy

The efficacy parameters assessed were change in A1C, body weight, SBP, and DBP from baseline in patients treated with canagliflozin 100 mg, canagliflozin 300 mg, or maximally tolerated glimepiride [21, 22]. At Week 52, both canagliflozin doses achieved reductions in all four efficacy parameters, and changes were maintained at Week 104. Maximally tolerated glimepiride achieved reductions in A1C at both Week 52 and Week 104, but had no effect on SBP and DBP, and a slight increase in body weight (Table 3).

**Table 3:** Efficacy parameters at Week 52 and Week 104 in the mITT analysis set [21, 22]

		CANA 100 mg (*N* = 483)	CANA 300 mg (*N* = 485)	GLIM (*N* = 482)
		LS mean change (SE)	LS mean change (SE)	LS mean change (SE)
A1C, %	52 weeks	−0.82 (0.04)	−0.93 (0.04)	−0.81 (0.04)
	104 weeks	−0.65 (0.04)	−0.74 (0.04)	−0.55 (0.04)
SBP, mm Hg	52 weeks	−3.3 (0.6)	−4.6 (0.6)	0.2 (0.6)
	104 weeks	−2.0 (0.6)	−3.1 (0.6)	1.7 (0.6)
DBP, mm Hg	52 weeks	−1.8 (0.4)	−2.5 (0.4)	−0.1 (0.4)
	104 weeks	−1.3 (0.4)	−2.2 (0.4)	−0.0 (0.4)
BW, kg	52 weeks	−3.7 (0.2)	−4.0 (0.2)	0.7 (0.2)
	104 weeks	−3.6 (0.2)	−3.6 (0.2)	0.8 (0.2)

*A1C* glycated hemoglobin, *BW* body weight, *CANA* canagliflozin, *DBP* diastolic blood pressure, *GLIM* glimepiride, *LS* least squares, *mITT* modified intent-to-treat, *SBP* systolic blood pressure, *SE* standard error

#### Attainment of QMs

Diabetes-related QM attainment at baseline, 52 weeks, and 104 weeks according to treatment arm is reported in Table 4. In addition to the proportion of patients achieving each measure, differences between treatment groups and the associated 95 % CI are represented. At baseline, the proportions of patients attaining QMs were generally similar across treatment groups.

**Table 4:** QMs Attainment at Baseline, Week 52 and Week 104 in the overall population

		CANA 100 mg (*N* = 483)		CANA 300 mg (*N* = 485)		GLIM (*N* = 482)
Measure		%	Difference vs. GLIM, % (95 % Cl)	%	Difference vs. GLIM, % (95 % Cl)	%
A1C						
<7.0 %	Baseline	12.2		14.6		13.5
	52 weeks	53.6	−2.3 (−8.8; 4.3)	60.1	4.3 (−2.2; 10.8)	55.7
	104 weeks	42.5	−1.4 (−7.9; 5.1)	50.2	6.3 (−0.2; 12.9)	43.9
<8.0 % (good control)	Baseline	61.1		59.9		60.1
	52 weeks	88.7	4.3 (−0.2; 8.9)	89.2	4.9 (0.4; 9.4)	84.4
	104 weeks	83.9	7.9 (2.7; 13.2)	84.6	8.6 (3.4; 13.9)	75.9
>9.0 % (poor control)	Baseline	6.5		7.2		7.2
	52 weeks	1.9	−2.1 (−4.5; 0.2)	1.7	−2.3 (−4.7; −0.0)	4.0
	104 weeks	2.5	−2.8 (−5.4; −0.1)	1.9	−3.4 (−5.9; −0.8)	5.3
Blood pressure						
<140/90 mm Hg	Baseline	74.1		74.2		75.0
	52 weeks	83.7	10.6 (5.2; 16.0)	85.4	12.3 (7.0; 17.6)	73.1
	104 weeks	79.6	5.4 (−0.1; 10.9)	84.2	10.0 (4.7; 15.3)	74.2
Body mass index and body weight						
≤30 kg/m^2^	Baseline	44.5		46.3		48.1
	52 weeks	57.4	10.5 (4.0; 17.1)	55.6	8.8 (2.2; 15.3)	46.9
	104 weeks	57.3	10.2 (3.7; 16.7)	54.8	7.7 (1.2; 14.2)	47.1
≥25 kg/m^2^ but ≥10 lb (4.5 kg) weight loss from baseline	Baseline					
	52 weeks	27.1	20.2 (15.4; 25.0)	34.6	27.7 (22.6; 32.7)	6.9
	104 weeks	27.9	20.0 (15.1; 24.9)	34.6	26.6 (21.5; 31.7)	7.9

*A1C* glycated hemoglobin, *CANA* canagliflozin, *GLIM* glimepiride, *95 % Cl* 95 % confidence interval

#### Canagliflozin 100 mg or 300 mg vs. glimepiride at 52 weeks

At 52 weeks, there was an increase in the percentage of patients with good glycemic control (A1C <7.0 %, <8.0 %), and a decrease in the percentage of those with poor glycemic control (A1C >9.0 %) (Table 4).

**Fig. 1:**
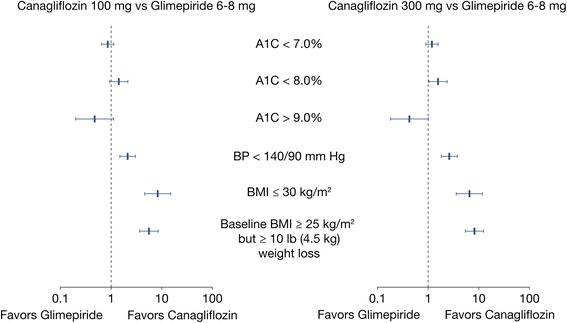
Odds ratio (95 % CIs) of canagliflozin 100 mg vs. glimepiride and canagliflozin 300 mg vs. glimepiride in the proportion of patients achieving QMs at Week 52. Mean (SD) maximum dose of GLIM was 5.6 (2.3) mg. CANA, canagliflozin; GLIM, glimepiride; QMs, quality measures; SD, standard deviation; 95 % Cl, 95 % confidence interval

Similar proportions of patients attained A1C <7.0 % across the treatment groups, with 53.6 % of canagliflozin 100 mg, 60.1 % of canagliflozin 300 mg, and 55.7 % of glimepiride treated patients (canagliflozin 100 mg vs. glimepiride OR = 0.85 [95 % CI 0.65; 1.13]; canagliflozin 300 mg vs. glimepiride OR = 1.18 [95 % CI 0.89; 1.56]). 4.3 % more patients treated with canagliflozin 100 mg achieved A1C <8.0 %; however, the CI for the difference with glimepiride included 0 (95 % CI -0.2; 8.9), with a corresponding OR of 1.42 [95 % CI 0.94; 2.14]. 4.9 % more patients treated with canagliflozin 300 mg (95 % CI 0.4; 9.4) attained this QM compared with glimepiride, with a corresponding OR of 1.57 (95 % CI 1.04; 2.37). No major differences were observed in the proportion of patients with A1C >9 % between canagliflozin 100 mg, canagliflozin 300 mg and glimepiride (the 95 % CIs for between-group differences included 0 and the 95 % CIs for ORs included 1) (Table 4 and Fig. 1).

Attainment of the measures related to BP and BMI/body weight reduction favored both canagliflozin doses compared with glimepiride at 52 weeks (Table 4 and Fig. 1). 10.6 % more patients with canagliflozin 100 mg (95 % CI 5.2 %; 16.0 %) and 12.3 % more patients with canagliflozin 300 mg (95 % CI 7.0 %; 17.6 %) attained BP <140/90 mm Hg vs. glimepiride; (Fig. 1). Similarly, 10.5 % more patients with canagliflozin 100 mg (95 % CI 4.0 %; 17.1 %), and 8.8 % more patients with canagliflozin 300 mg (95 % CI 2.2 %; 15.3 %) achieved BMI ≤30 kg/m^2^ compared with glimepiride. Composite measure of ≥25 kg/m^2^ and ≥10 lb (4.5 kg) weight loss from baseline also favored canagliflozin treatment groups (Table 4 and Fig. 1). There was a 20.2 % (95 % CI 15.4 %; 25.0 %) and 27.7 % (95 % CI 22.6 %; 32.7 %) increase in the proportion of patients achieving this composite QM in the canagliflozin 100 mg and canagliflozin 300 mg groups, respectively, compared with glimepiride. The 95 % CIs for the ORs reflecting on-treatment differences after 52 weeks excluded 1 for each BP and BMI/body weight-related quality measure.

#### Canagliflozin 100 mg or 300 mg vs. glimepiride at 104 weeks

At 104 weeks, in all three treatment groups, the proportion of patients attaining glycemic measures of A1C <7.0 % and <8.0 % increased from baseline, while the proportion of patients with poor glycemic control (A1C >9.0 %) decreased from baseline.

**Fig. 2:**
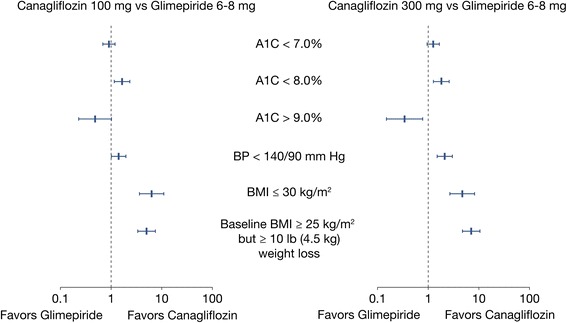
Odds ratio (95 % CIs) of canagliflozin 100 mg vs. glimepiride and canagliflozin 300 mg vs. glimepiride in the proportion of patients achieving QMs at Week 104. Mean (SD) maximum dose of GLIM was 5.8 (2.2) mg. CANA, canagliflozin; GLIM, glimepiride; QMs, quality measures; SD, standard deviation; 95 % Cl, 95 % confidence interval

Both doses of canagliflozin demonstrated similar attainment of A1C <7.0 % compared with glimepiride, with 42.5 % of canagliflozin 100 mg, 50.2 % of canagliflozin 300 mg, and 43.9 % of glimepiride-treated patients (canagliflozin 100 mg vs. glimepiride OR = 0.89 [95 % CI 0.68; 1.18]; canagliflozin 300 mg vs. glimepiride OR = 1.29 [95 % CI 0.98; 1.70]) (Table 4 and Fig. 2). 7.9 % more patients with canagliflozin 100 mg (95 % CI 2.7 %; 13.2 %) attained A1C <8.0 % at Week 104 than glimepiride, with a corresponding OR of 1.64 (95 % CI 1.16; 2.33); while 8.6 % more patients treated with canagliflozin 300 mg (95 % CI 3.4 %; 13.9 %) achieved the same QM with an OR of 1.82 (95 % CI 1.27; 2.59). The proportion of patients with A1C >9.0 % was 3.4 % lower for canagliflozin 300 mg (95 % CI −5.9 %; −0.8 %) than for glimepiride with a corresponding OR of 0.35 (95 % CI 0.15; 0.78), but similar when comparing canagliflozin 100 mg with glimepiride (OR = 0.48 [95 % CI 0.23; 1.02]).

Attainment of QMs related to BP favored canagliflozin (Table 4 and Fig. 2); a higher percentage of patients had a BP <140/90 mm Hg in the canagliflozin 100 mg (5.4 %, 95 % CI −0.1 %; 10.9 %) and canagliflozin 300 mg (10.0 %, 95 % CI 4.7 %; 15.3 %) groups compared with glimepiride with ORs of 1.41 (95 % CI 1.01; 1.96) and 2.12 (95 % CI 1.49; 3.00), respectively.

Similarly, attainment of both BMI/body weight reduction measures favored canagliflozin compared with glimepiride at 104 weeks (Table 4 and Fig. 2). 10.2 % more patients who received canagliflozin 100 mg had a BMI ≤30 kg/m^2^ at 104 weeks compared with glimepiride, with a corresponding OR of 6.32 (95 % CI 3.64; 10.98), while 7.7 % more patients who received canagliflozin 300 mg (95 % CI 1.2 %; 14.2 %) attained this QM compared with glimepiride (OR = 4.70 [95 % CI 2.68; 8.24]). The composite measure of ≥25 kg/m^2^ and ≥10 lb (4.5 kg) weight loss from baseline also favored canagliflozin treatment at 104 weeks, with 20.0 % (95 % CI 15.1 %; 24.9 %, OR = 5.01 [95 % CI 3.36; 7.49]) and 26.6 % (95 % CI 21.5 %; 31.7 %, OR = 7.06 [95 % CI 4.75; 10.49]) more patients in the canagliflozin 100 mg and 300 mg groups respectively, compared with glimepiride.

#### Safety analysis

A summary of selected AEs for the core and entire treatment periods is presented in Table 5. As previously reported [20, 21], the total number of AEs was similar between groups, while serious AEs tended to occur more frequently with glimepiride. The number of major adverse cardiovascular events and events of hospitalized unstable angina (MACE-plus) were low across all treatment groups, with no increase in MACE-plus associated risk with canagliflozin use.

**Table 5:** Summary of selected AEs in the overall population

	Baseline – 52 weeks			Baseline – 104 weeks		
	CANA 100 mg (*N* = 483)	CANA 300 mg (*N* = 485)	GLIM (*N* = 482)	CANA 100 mg (*N* = 483)	CANA 300 mg (*N* = 485)	GLIM (*N* = 482)
	n (%)	n (%)	n (%)	n (%)	n (%)	n (%)
Any AEs	311 (64.4)	332 (68.5)	330 (68.5)	354 (73.3)	378 (77.9)	378 (78.4)
AEs leading to discontinuation	25 (5.2)	32 (6.6)	28 (5.8)	30 (6.2)	46 (9.5)	35 (7.3)
Serious AEs	24 (5.0)	26 (5.4)	39 (8.1)	47 (9.7)	47 (9.7)	69 (14.3)
Serious AEs leading to discontinuation	6 (1.2)	5 (1.0)	10 (2.1)	8 (1.7)	8 (1.6)	13 (2.7)
Cardiovascular AEs						
Cardiovascular death	2 (0.4)	0	1 (0.2)	5 (1.0)	0	2 (0.4)
Nonfatal myocardial infarction	2 (0.4)	1 (0.2)	1 (0.2)	4 (0.8)	3 (0.6)	4 (0.8)
Nonfatal stroke	2 (0.4)	2 (0.4)	0	2 (0.4)	3 (0.6)	2 (0.4)
Hospitalized unstable angina	0	1 (0.2)	1 (0.2)	1 (0.2)	1 (0.2)	1 (0.2)
MACE-plus	5 (1.0)	4 (0.8)	3 (0.6)	10 (2.1)	7 (1.4)	9 (1.9)
Patients with any documented hypoglycemia^a^	27 (5.6)	24 (4.9)	165 (34.2)	33 (6.8)	40 (8.2)	197 (40.9)
Biochemically documented hypoglycemia	26 (5.4)	21 (4.3)	164 (34.0)	32 (6.6)	39 (8.0)	197 (40.9)
Severe hypoglycemia	2 (0.4)	3 (0.6)	15 (3.1)	3 (0.6)	1 (0.2)	16 (3.3)
Total number of episodes of documented hypoglycemia^a^	67	33	710	106	100	1189
Patients with documented hypoglycemia episodes, n (%)^a^						
1 episode	11 (2.3)	17 (3.5)	45 (9.3)	15 (3.1)	24 (4.9)	44 (9.1)
2 episodes	7 (1.4)	5 (1.0)	32 (6.6)	7 (1.4)	8 (1.6)	34 (7.1)
≥3 episodes	9 (1.9)	2 (0.4)	88 (18.3)	11 (2.3)	8 (1.6)	119 (24.7)
Genital mycotic infections						
Male	17 (6.7)	20 (8.3)	3 (1.1)	24 (9.5)	22 (9.1)	5 (1.9)
Female	26 (11.3)	34 (13.9)	5 (2.3)	32 (13.9)	38 (15.6)	6 (2.7)
Urinary tract infections	31 (6.4)	31 (6.4)	22 (4.6)	51 (10.6)	42 (8.7)	33 (6.8)
Osmotic diuresis-related AEs	27 (5.6)	30 (6.2)	8 (1.7)	28 (5.8)	32 (6.6)	10 (2.1)
Pollakiuria	12 (2.5)	12 (2.5)	1 (0.2)	13 (2.7)	12 (2.5)	2 (0.4)
Polyuria	4 (0.8)	4 (0.8)	2 (0.4)	4 (0.8)	5 (1.0)	3 (0.6)
Volume depletion AEs	8 (1.7)	9 (1.9)	8 (1.7)	8 (1.7)	12 (2.5)	11 (2.3)

^a^Episodes of hypoglycemia are prior to rescue medication; other AEs are regardless of rescue medication. *AEs* adverse events, *CANA* canagliflozin, *GLIM* glimepiride, *MACE-plus* major adverse cardiovascular events and events of hospitalized unstable angina

The total number of documented hypoglycemia episodes, the number of patients with any documented hypoglycemia, and the number of patients with biochemically documented hypoglycemia, or severe hypoglycemia, were lower with canagliflozin 100 mg and 300 mg than with glimepiride. A lower incidence of documented hypoglycemia during both the core and entire treatment periods was observed with canagliflozin compared with glimepiride (baseline through 52 weeks: 5.6 %, 4.9 %, and 34.2 % with canagliflozin 100 mg, 300 mg and glimepiride, respectively; baseline through 104 weeks: 6.8 %, 8.2 %, and 40.9 % with canagliflozin 100 mg, 300 mg, and glimepiride, respectively). The total numbers of episodes of documented hypoglycemia with canagliflozin 100 mg, 300 mg, and glimepiride were 67, 33, and 710 during the core period, and 106, 100, and 1,189 during the entire treatment period, respectively.

During both the core and entire treatment periods, the incidence of GMIs in both male and female patients was higher in those treated with canagliflozin 100 mg and 300 mg compared with glimepiride (baseline through 52 weeks in males: 6.7 %, 8.3 %, and 1.1 %; in females: 11.3 %, 13.9 %, and 2.3 %, respectively; baseline through 104 weeks in males: 9.5 %, 9.1 %, and 1.9 %; in females: 13.9 %, 15.6 %, and 2.7 %, respectively). Similarly, patients treated with canagliflozin 100 mg and 300 mg had a higher incidence of UTIs compared with glimepiride (baseline through 52 weeks: 6.4 %, 6.4 %, and 4.6 %; baseline through 104 weeks: 10.6 %, 8.7 %, and 6.8 %, with canagliflozin 100 mg, 300 mg and glimepiride, respectively), as well as a higher incidence of osmotic-diuresis-related AEs (baseline through 52 weeks: 5.6 %, 6.2 %, and 1.7 %; baseline through 104 weeks: 5.8 %, 6.6 %, and 2.1 %, respectively). The incidence of volume depletion AEs was low and comparable across treatment groups (baseline through 52 weeks: 1.7 %, 1.9 %, and 1.7 %; baseline through 104 weeks: 1.7 %, 2.5 %, and 2.3 % with canagliflozin 100 mg, 300 mg, and glimepiride, respectively). There was no reported serious case of diabetic ketoacidosis (DKA) related to canagliflozin use.

Similar to the results of other Phase 3 trials of SGLT2 inhibitors approved for use in the US (canagliflozin, dapagliflozin and empagliflozin), canagliflozin was associated with an increase in LDL-C levels in the CANTATA-SU trial [24–26]. The mean percent change in LDL-C increased from baseline to Week 26, and remained stable through Week 52 in all treatment groups; LS mean change (SE) from baseline at Week 52 was 9.6 (1.9) for canagliflozin 100 mg, 14.1 (1.9) for canagliflozin 300 mg, and 5.0 (1.9) for glimepiride. There was no increase in the risk of bone fractures with canagliflozin use; the number of subjects with fracture AEs was 12 in canagliflozin 100 mg, and 13 in canagliflozin 300 mg and glimepiride groups. Across treatment groups, ≤10 % of subjects initiated or dose-adjusted for common antihypertensive agents. More subjects in the glimepiride group than in the canagliflozin groups initiated or had dose-adjusted common antihypertensive agents (β-blockers, agents acting on the renin-angiotensin system, and calcium-channel blockers). The proportion of patients using other antihypertensive agents in the mITT population was higher at 104 weeks than at baseline in the glimepiride group (2.5 % vs. 1.5 %) but remained similar in the canagliflozin 100 mg group (2.5 % vs. 2.1 %) and canagliflozin 300 mg group (3.1 % vs. 2.7 %).

## Discussion

Diabetes-related QMs are becoming increasingly important as indicators of quality of diabetes care. Our study showed a better attainment of diabetes-related measures with canagliflozin compared with glimepiride. Both doses of canagliflozin as well as glimepiride led to a decrease in A1C values compared with baseline; however, canagliflozin 100 mg resulted in similar or greater attainment of QMs related to glycemic control and BP, while canagliflozin 300 mg resulted in superior attainment of QMs related to glycemic control and BP compared with glimepiride.

The Eighth Joint National Committee recommends a BP target <140/90 mm Hg in diabetic patients, since reductions in BP in patients with diabetes who suffer from hypertension can improve cardiovascular health and mortality [27]. Our study showed that significantly more patients receiving both doses of canagliflozin achieved this target compared with glimepiride. Furthermore, this QM was attained even though higher proportions of patients in the glimepiride group were receiving antihypertensive agents.

Both canagliflozin doses resulted in more overweight or obese patients achieving weight loss. Weight gain in patients with T2DM has been shown to lead to patient frustration and can have a negative impact on medication adherence [28]. Conversely, weight loss in patients with T2DM has been associated with improved medication adherence, leading to the suggestion that diabetes medications that promote weight loss may be beneficial in this regard [29]. Other studies in obese patients with T2DM have demonstrated positive effects on psychological well-being and health-related quality of life as a result of achieving weight loss [30]. This post-hoc analysis showed that patients treated with canagliflozin 100 mg or 300 mg had larger decreases in body weight at 52 weeks compared with glimepiride, which were also maintained at 104 weeks. Additionally, among the patient subgroup with a BMI ≥25 kg/m^2^, a greater proportion of patients in both canagliflozin groups, compared with the glimepiride group, were found to have lost ≥10 lb (4.5 kg) of body weight at 52 and 104 weeks. These results may indicate a beneficial effect of canagliflozin compared with maximally tolerated glimepiride in the achievement of diabetes QMs related to body weight. Although prevention of hypoglycemia is not a QM, there was a marked reduction in numbers of episodes when comparing both doses of canagliflozin with glimepiride, which might have further contributed to the weight benefits.

Similarly to the study presented here, a previous post-hoc analysis compared the two doses of canagliflozin (100 mg and 300 mg) with the dipeptidyl peptidase-4 (DPP-4) inhibitor sitagliptin (100 mg) in the attainment of diabetes-related QMs of glycemic control, BP, and body weight. Compared with sitagliptin, canagliflozin 100 mg was associated with comparable or superior attainment of QMs, and canagliflozin 300 mg was associated with superior attainment of all QMs [20], which shows a consistent benefit of canagliflozin over different AHAs.

Safety results of the CANTATA-SU trial indicate that canagliflozin is well-tolerated, with GMI and UTIs as the most common drug-related AEs. There have been reports of ketoacidosis in patients with type 1 diabetes and T2DM treated with SGLT2 inhibitors [31–33]; however, no serious incident of DKA related to canagliflozin was reported in this study. Erondu et al. recently analyzed all serious AEs of DKA and related events in 17,596 patients from randomized clinical trials of canagliflozin. Their findings support that the incidence of DKA and related events is rare with canagliflozin use (0.07 %) [34]. Similarly, the frequency of reported events of DKA was found to be low (<0.1 %) in randomized controlled trials involving SGLT2 inhibitors dapagliflozin (preliminary) and empagliflozin [35, 36].

Previous studies have shown that the use of SGLT2 inhibitors is associated with changes in lipid profiles [24, 26, 37] Pooled analyses of placebo-controlled Phase 3 trials of canagliflozin, dapagliflozin, and empagliflozin reported small increases in LDL-C and high-density lipoprotein-cholesterol, and small decreases in triglyceride levels from baseline [24–26]. Similarly in the CANTATA-SU trial, there was a small increase from baseline in the mean percent change in LDL-C levels with canagliflozin use. However, the clinical relevance of these changes in patients’ lipid profiles is not clear and LDL-C treatment targets have been removed from the ACC/AHA Task Force on Practice Guidelines and HEDIS diabetes-related QMs [15]. Findings from the EMPA-REG OUTCOME study have shown that empagliflozin provides cardiovascular benefits in patients with T2DM at high risk for cardiovascular events [36]. Furthermore, a recent meta-analysis on the effects of SGLT2 inhibitors on cardiovascular events, death, and safety outcomes in adults with T2DM has suggested net protection of SGLT2 inhibitors against cardiovascular outcomes and death [38].

Patients with T2DM were shown to have a higher risk of fractures, which increases with advancing age and might be associated with the use of specific AHAs [39]. In a pool of 9 clinical trials, Watts et al. recently evaluated the occurrence of bone fractures with canagliflozin treatment and found that the incidence of fractures was similar in canagliflozin (1.7 %) and non-canagliflozin (1.5 %) groups in patients with no prior history/risk of cardiovascular disease [40]. Similarly in this study, no increase in the risk of fractures with canagliflozin use was observed.

The use of QMs is becoming increasingly important in the evaluation of the quality of care across healthcare delivery systems. For Accountable Care Organizations that participate in the Medicare Shared Savings Program, quality benchmarks have recently been established and the performance of participating organizations is judged against specific QMs before shared payments are made [41]. QMs also provide a foundation for initiatives such as Pay-for-Performance (P4P), which aim to reward physicians and non-physician clinicians for improvements in quality of care [42]. Since many patients with T2DM have hypertension, P4P programs not only reward the achievement of glycemic goals, but also goals associated with other parameters including BP [43].

A particular strength of this study is its use of data from a clinical trial that compared the efficacy and safety of canagliflozin with glimepiride, an active comparator drug. Other trials have compared a sulfonylurea with newer AHAs such as a DPP-4 inhibitor [44] or SGLT2 inhibitors dapagliflozin and empagliflozin [45, 46]. However, these studies limited the up-titration of the sulfonylurea to the initial 12 to 18 weeks of the study, thereby leading to a sub-optimal and less-clinically relevant comparison. The sub-optimal titration may drive the outcome to lower decreases in A1C for the sulfonylurea treatment group. In contrast, in the CANTATA-SU study, the sulfonylurea glimepiride was allowed to be up-titrated throughout the entire study period (104 weeks with a 52-week primary endpoint time point) and did not have a fixed duration of titration. This resulted in a mean final dose achieved of 5.8 mg, allowing for a more relevant comparison with the study drug [22]. The A1C range specified in the patient eligibility criteria for inclusion in the clinical trial may limit the generalizability of the findings of this analysis to patients with more severe hyperglycemia (patients with A1C >9.5 % were not included in the trial [22]).

## Conclusions

In this analysis of data from the randomized, double-blind, Phase 3 CANTATA-SU trial, using the recently revised and currently accepted diabetes-related QMs, canagliflozin provided superior glycemic control compared with glimepiride in patients with T2DM who were poorly controlled on metformin monotherapy. Compared with glimepiride, canagliflozin resulted in improvement in QMs related to BP and weight loss, and was additionally associated with fewer incidences of hypoglycemic events. These observations on diabetes-related QM attainment may be useful to those making decisions at the population level.

## Abbreviations

A1C, glycated hemoglobin; AACE, American Association of Clinical Endocrinologists; ACE, American College of Endocrinology; ADA, American Diabetes Association; AE, adverse event; AHA, antihyperglycemic agent; ANCOVA, analysis of covariance; BMI, body mass index; BP, blood pressure; BW, body weight; CANA, canagliflozin; CANTATA-SU, CANagliflozin Treatment And Trial Analysis Sulfonyl Urea; DBP, diastolic blood pressure; DPP-4, dipeptidyl peptidase-4; EASD, European Association for the Study of Diabetes; eGFR, estimated glomerular filtration rate; GLIM, glimepiride; GMI, genital mycotic infection; HEDIS, Healthcare Effectiveness Data and Information Set; LDL-C, low-density lipoprotein-C; LOCF, last observation carried forward; LS, least squares; mITT, modified intent-to-treat; P4P, pay-for-performance; QM, quality measure; SBP, systolic blood pressure; SD, standard deviation; SE, standard error; SGLT2, sodium-glucose co-transporter 2; T2DM, type 2 diabetes mellitus; US, United States; UTI, urinary tract infection
